# The Fluctuations of Leukocytes and Circulating Cytokines in Septic Humanized Mice Vary With Outcome

**DOI:** 10.3389/fimmu.2019.01427

**Published:** 2019-06-26

**Authors:** Tomasz Skirecki, Susanne Drechsler, Grazyna Hoser, Mohammad Jafarmadar, Katarzyna Siennicka, Zygmunt Pojda, Jerzy Kawiak, Marcin F. Osuchowski

**Affiliations:** ^1^Laboratory of Flow Cytometry, Centre of Postgraduate Medical Education, Warsaw, Poland; ^2^Ludwig Boltzmann Institute for Experimental and Clinical Traumatology in the AUVA Research Center, Vienna, Austria; ^3^Department of Regenerative Medicine, Maria Sklodowska-Curie Institute-Oncology Center, Warsaw, Poland

**Keywords:** sepsis, humanized mouse, cecal ligation and puncture (CLP), peritonitis, outcome prediction, immunity

## Abstract

Sepsis remains a major challenge in translational research given its heterogeneous pathophysiology and the lack of specific therapeutics. The use of humanized mouse chimeras with transplanted human hematopoietic cells may improve the clinical relevance of pre-clinical studies. However, knowledge of the human immuno-inflammatory response during sepsis in humanized mice is scarce; it is unclear how similar or divergent mouse and human-origin immuno-inflammatory responses in sepsis are. In this study, we evaluated the early outcome-dependent immuno-inflammatory response in humanized mice generated in the NSG strain after cecal ligation and puncture (CLP) sepsis. Mice were observed for 32 h post-CLP and were assigned to either predicted-to-die (P-DIE) or predicted-to-survive (P-SUR) groups for retrospective comparisons. Blood samples were collected at baseline, 6 and 24 h, whereas the bone marrow and spleen were collected between 24 and 32 h post-CLP. In comparison to P-SUR, P-DIE humanized mice had a 3-fold higher frequency of human splenic monocytes and their CD80 expression was reduced by 1.3-fold; there was no difference in the HLA-DR expression. Similarly, the expression of CD80 on the bone marrow monocytes from P-DIE mice was decreased by 32% (*p* < 0.05). Sepsis induced a generalized up-regulation of both human and murine plasma cytokines (TNFα, IL-6, IL-10, IL-8/KC, MCP-1); it was additionally aggravated in P-DIE vs. P-SUR. Human cytokines were strongly overridden by the murine ones (approx. ratio 1:9) but human TNFα was 7-fold higher than mouse TNFα. Interestingly, transplantation of human cells did not influence murine cytokine response in NSG mice, but humanized NSG mice were more susceptible to sepsis in comparison with NSG mice (79 vs. 33% mortality; *p* < 0.05). In conclusion, our results show that humanized mice reflect selected aspects of human immune responses in sepsis and therefore may be a feasible alternative in preclinical immunotherapy modeling.

## Introduction

In more than 30 years of intensive research no new specific therapeutics for sepsis have been introduced into clinical care (1). Despite improved standards of care, the mortality rates of sepsis have not decreased (2). On the contrary, prevalence has been on the rise, reaching approximately 19 million sepsis cases per year worldwide (3), making sepsis a global medical burden (4).

The failure of anti-sepsis therapeutics to modulate the host response has been attributed to a large part to flawed pre-clinical studies (5–7). Currently, a vast majority of sepsis animal studies are performed in mice (8); one of the major translational obstacles in murine models of sepsis are substantial mouse-human differences in the immune system (9). The development of humanized mice is emerging as a promising platform for at least partially overcoming those disparities. Humanized mice are generated by xenotransplantation of human hematopoietic stem cells (HSCs) into immunocompromised mice which were additionally irradiated or chemoablated to empty their bone marrow niches for the xenograft (10). Currently, one of the most commonly used mouse strains for humanization is the NSG (NOD scid gamma) strain (11); NSG mice have composite immunodeficiency given that they lack not only lymphocytes, NK cells, and macrophages but also have an impaired complement system, enabling an efficient engraftment of the human cells with a relatively low risk of developing graft-vs.-host disease.

Humanized mice have already been used to model acute infectious diseases and have proven useful in both recapitulating some unique human responses as well as suggesting therapeutic solutions (12–14). On a limited scale, humanized mice were also employed in sepsis research using the cecal ligation and puncture (CLP), the gold standard model to recapitulate septic peritonitis (15–18). CLP-induced immuno-inflammatory responses vary according to the underlying pathophysiology, i.e., in a non-marginal severity CLP protocol (i.e., neither 100% mortality/survival), mice subjected to the same insult develop an individual response and progress to either survival or death without following a predefined immune “outcome pattern” from the onset of CLP (19). Outcomes in CLP mice can be predicted (e.g., mice predicted-to-survive/die) using body temperature and/or biomarker measurements as well as clinical assessment scores (19–22). Comparisons of such dynamic signaling changes between the surviving and dying phenotypes advance our understanding of mechanisms co-responsible for sepsis lethality (and recovery) better than simplistic (but uniformly statistically significant) comparisons of healthy to septic subjects (21–23). Humanized mice are an attractive platform for the investigation of human immuno-inflammatory responses (and treatments targeting these systems) in critical care diseases including sepsis. It is therefore crucial to characterize the cellular and cytokine responses, given that the complexity of the humanized sepsis mouse model is likely exacerbated by various inter-species interactions (11). The concomitant presence of cells and mediators of both human and murine origin results in multi-directional crosstalk which likely alters the immune-inflammatory dynamics of the host in severe infections. It is largely unclear what action the transplanted human immune cells exert upon the mouse host's response to septic insult and how impactful this interaction can be. Such a knowledge gap hinders the utility of humanized mice in modeling of sepsis as well as the translation of findings generated in such models.

To partially address the above unknowns, we subjected humanized NSG mice to polymicrobial abdominal sepsis and investigated two specific questions: (a) comparison of the initial cellular and humoral inflammatory response of human and murine origin and (b) characterization of those responses depending on outcome (i.e., dying vs. surviving) in the early phase of sepsis.

## Methods

### Mice

Mice of the NSG strain (NOD.Cg-Prkdcscid Il2rgtm1Wjl/SzJ) and SCID strain were obtained from The Jackson Laboratories (Bar Harbour, ME, USA). Mice were bred in the animal facility of the Center of Postgraduate Medical Education (Warsaw, Poland) under pathogen-free conditions with 12/12 light cycle and fed with standard sterile diet and drinking water ad libitum. All experiments on animals were approved by the Local Ethics Committee no IV in Warsaw, Poland (92/2012) and adhered to the ARRIVE guidelines (24).

### Generation of Humanized Mice

#### Human Stem Cell Harvesting

To generate humanized mice, we followed the previously published protocol from our lab (17). In brief, human umbilical cord blood (UCB) probes were processed in accordance with the procedures approved by the Institutional Review Board of the Maria Sklodowska-Curie Memorial Cancer Center and Institute of Oncology (Warsaw, Poland). The UCB units from healthy donor mothers were obtained with their informed consent. Then, human HSCs were isolated from Ficoll-centrifuged mononuclear cell fraction of human UCB and stored in liquid nitrogen after isolation. CD34-positive human stem and progenitor cells were purified from thawed mononuclear cells by positive immunomagnetic separation technique using a commercially available kit (EasySep, Stemcell Technologies, Vancouver, BC, Canada). Purity of isolated cells (>90%) was tested by flow cytometry using anti-CD34 PE/anti-CD45 FITC antibodies (BD Bioscience, San Jose, CA, USA).

#### Transplantation

Three- to four-week-old NSG female mice were pretreated with i.p. injections of busulfan (25 mg/kg) (Sigma-Aldrich) for 2 consecutive days before stem cell transplantation. Female mice were chosen as they are known to present a higher engraftment rate (25). Twenty-four hours after the last busulfan dose, mice were given a tail vein injection of 10^5^ purified human UCB CD34+ cells. Seven weeks after transplantation, 20 μl of blood was obtained via facial vein puncture (23G needle). For the flow cytometry evaluation of human chimerism, the blood samples were stained with anti-human CD45 FITC (BD) antibody. A total of 33 mice were deemed as humanized (blood chimerism over 5%) and were enrolled in the CLP study.

### CLP Sepsis Model

#### Surgery and Treatment

In order to reproduce human peritonitis-derived sepsis, we performed the CLP surgery in 33 humanized mice according to the original protocol from Wichterman et al. (26) with modifications (22, 27). In brief, all mice received analgesia (i.e., buprenorphine, 0.05 mg/kg in 1 ml of 0.9% saline) 20 min prior to CLP and the abdomen was shaved and disinfected with alcohol. After midline laparotomy, the cecum was exposed, ligated underneath the ileocecal valve, and a needle puncture (through-and-through) was applied. After repositioning of the cecum, the abdomen was closed with single button sutures and Histoacryl® tissue adhesive (B. Braun, Aesculap, Germany).

**Figure 1:**
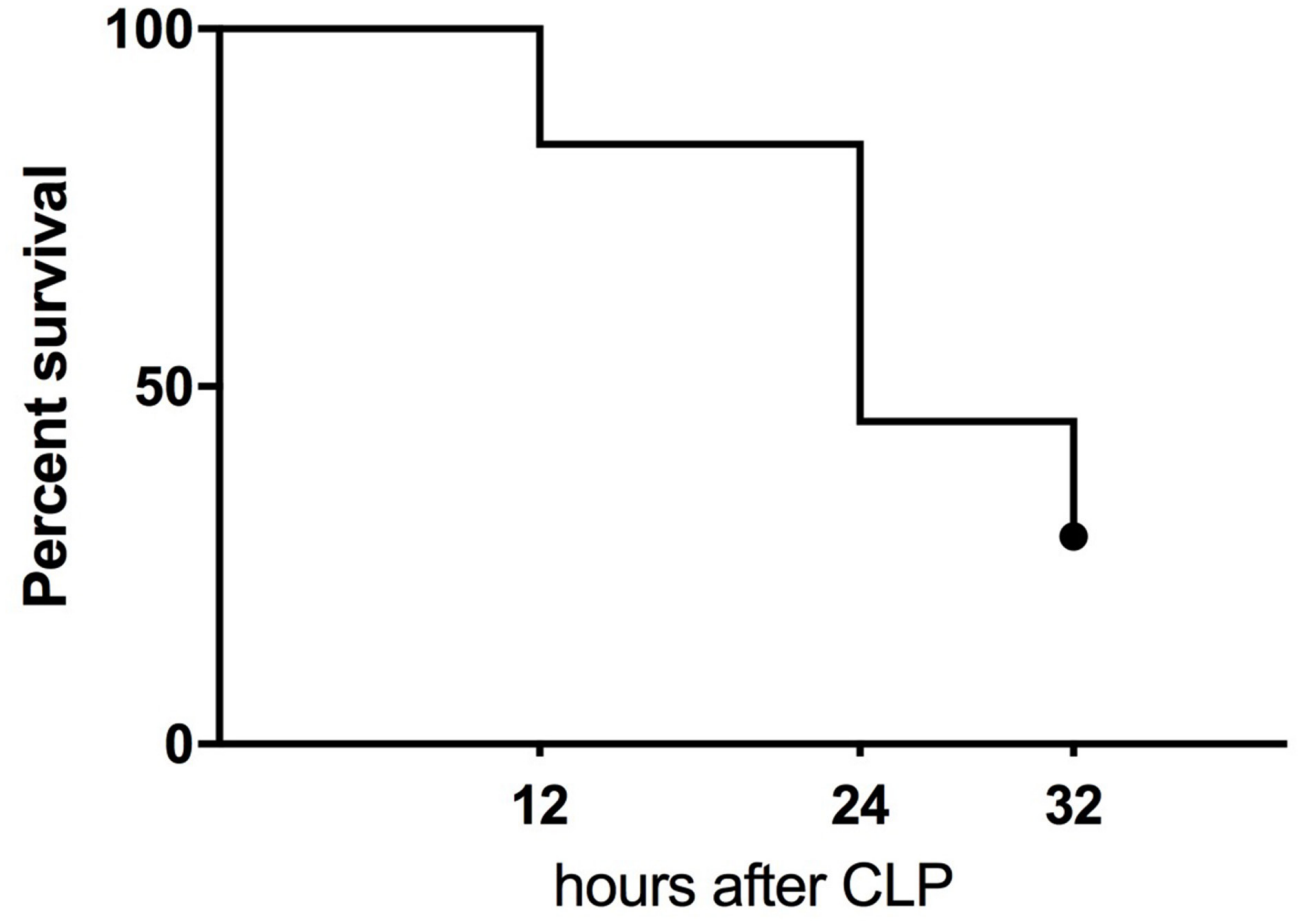
Kaplan-Meier survival curve of humanized NSG mice subjected to CLP. The combined mortality of CLP induced by double puncture with 25G needle (*n* = 19) and 27G (*n* = 14).

**Figure 2:**
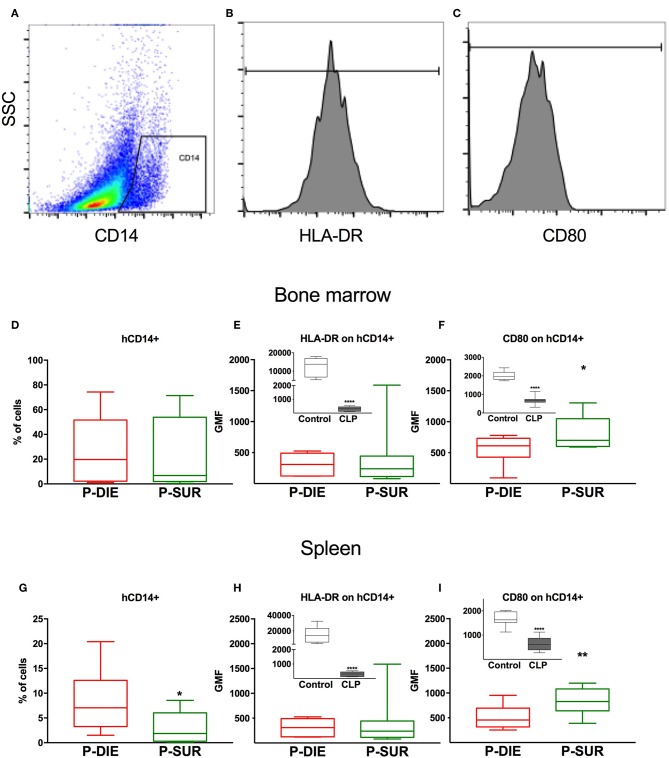
Human monocyte response regarding sepsis outcome. **(A)** Representative cytograms and histograms of the flow cytometry analysis of human CD14+ monocytes in the spleen and their **(B)** HLA-DR and **(C)** CD80 expression. Results of the analysis of bone marrow cells: **(D)** Frequency of human CD14+ monocytes. **(E)** Geometric mean fluorescence (GMF) of anti-HLA-DR staining of human monocytes. **(F)** GMF of anti-CD80 staining of human monocytes. Analysis of splenic cells: **(G)** Frequency of human monocytes. **(H)** GMF of anti-HLA-DR staining and **(I)** GMF of anti-CD80 staining of human monocytes. Insets present a comparison of healthy (*n* = 7) vs. all septic mice (*n* = 20; irrespective of outcome). Groups were compared using *t*-test. *p < 0.05; ** *p* < 0.01; ****p < 0.0001.

**Figure 3:**
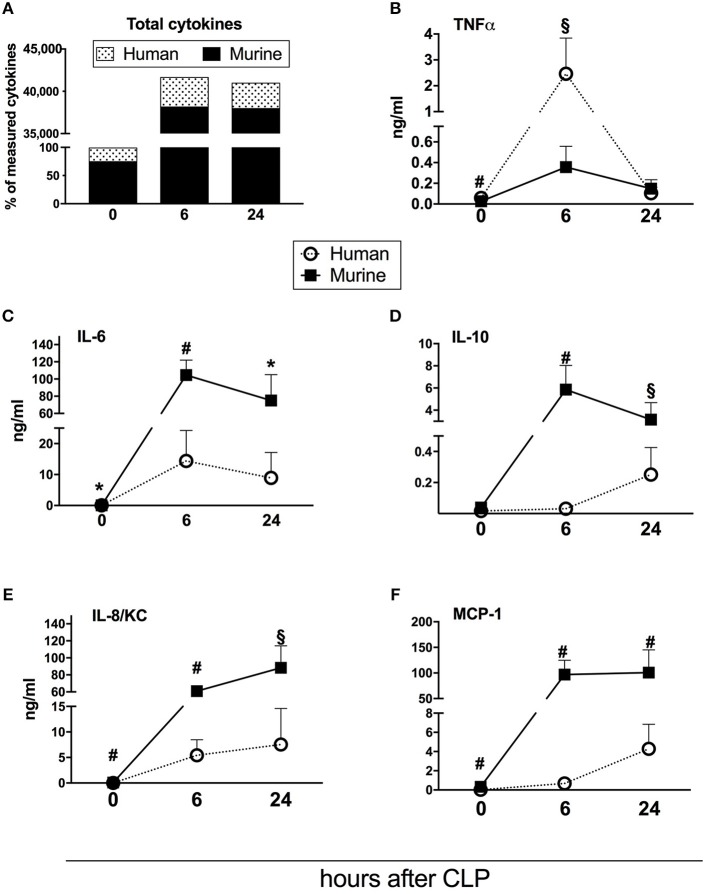
Dynamics of human and murine cytokine responses after CLP regardless of outcome. Graphs present concentration of cytokines from 31 humanized NSG mice subjected to CLP. Dynamics of: **(A)** combined murine and human cytokines (the sum of both measured at baseline counted as 100%); **(B)** TNF; **(C)** IL-6; **(D)** IL-10; **(E)** IL-8/KC; **(F)** MCP-1. BL, baseline. Concentration of human and murine cytokines were compared using Mann-Whitney test at each time-point separately (T0,T6h n = 31, T24 n = 19). *p < 0.05; §*p* < 0.01; #*p* < 0.001.

**Figure 4:**
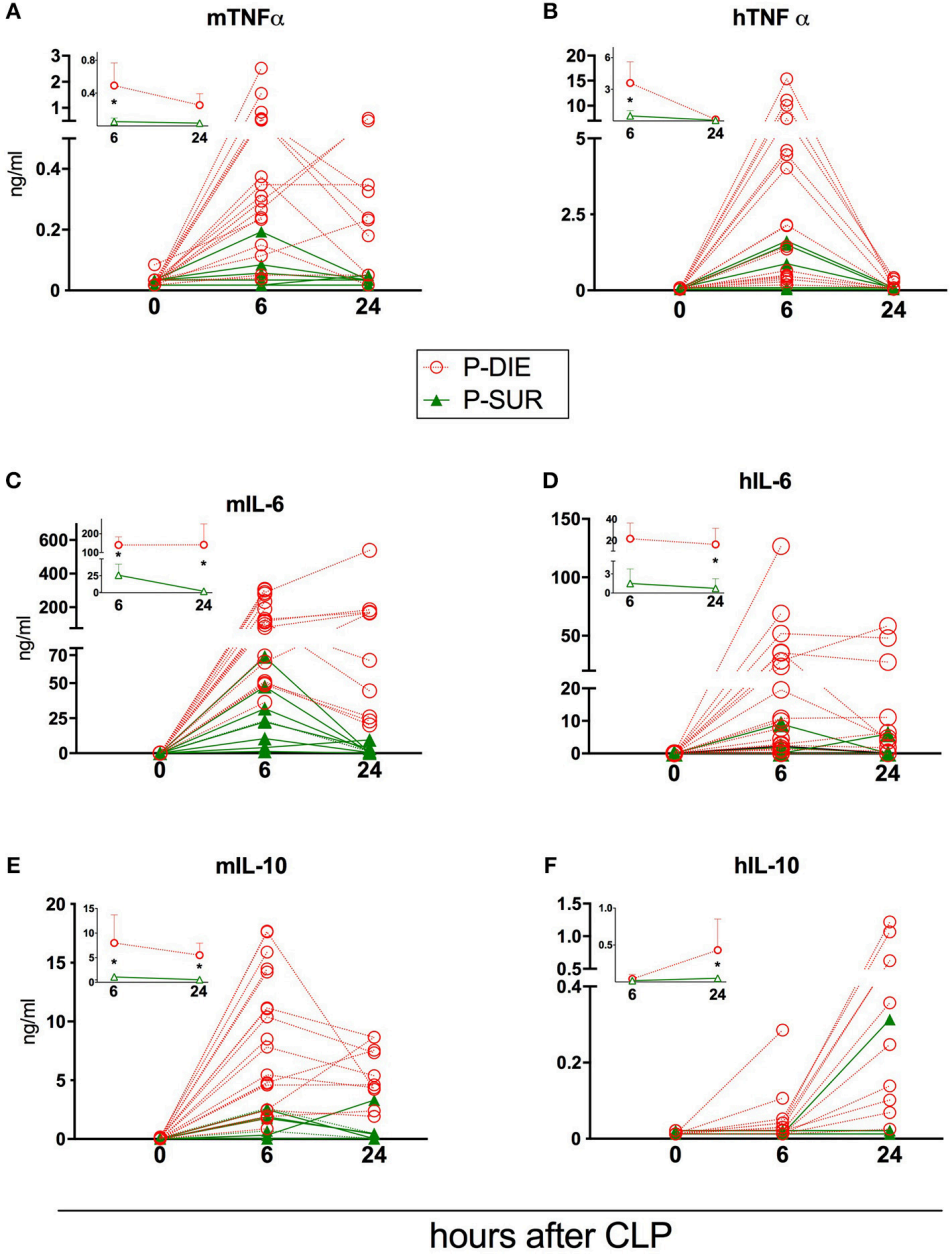
Comparison of human and murine cytokines depending on the post-CLP outcome in humanized mice. **(A)** murine TNF; **(B)** human TNF; **(C)** murine IL-6; **(D)** human IL-6; **(E)** murine IL-10; **(F)** human IL-10. (P-Die *n* = 23, P-Sur *n* = 8). Insets present a comparison of P-DIE (*n* = 22) vs. P-SUR mice (*n* = 9). Data are presented as mean values and 95% confidence interval bars. Concentrations of cytokines of P-SUR and P-DIE mice were compared by the *t*-test at each time-point separately. *p < 0.05.

**Figure 5:**
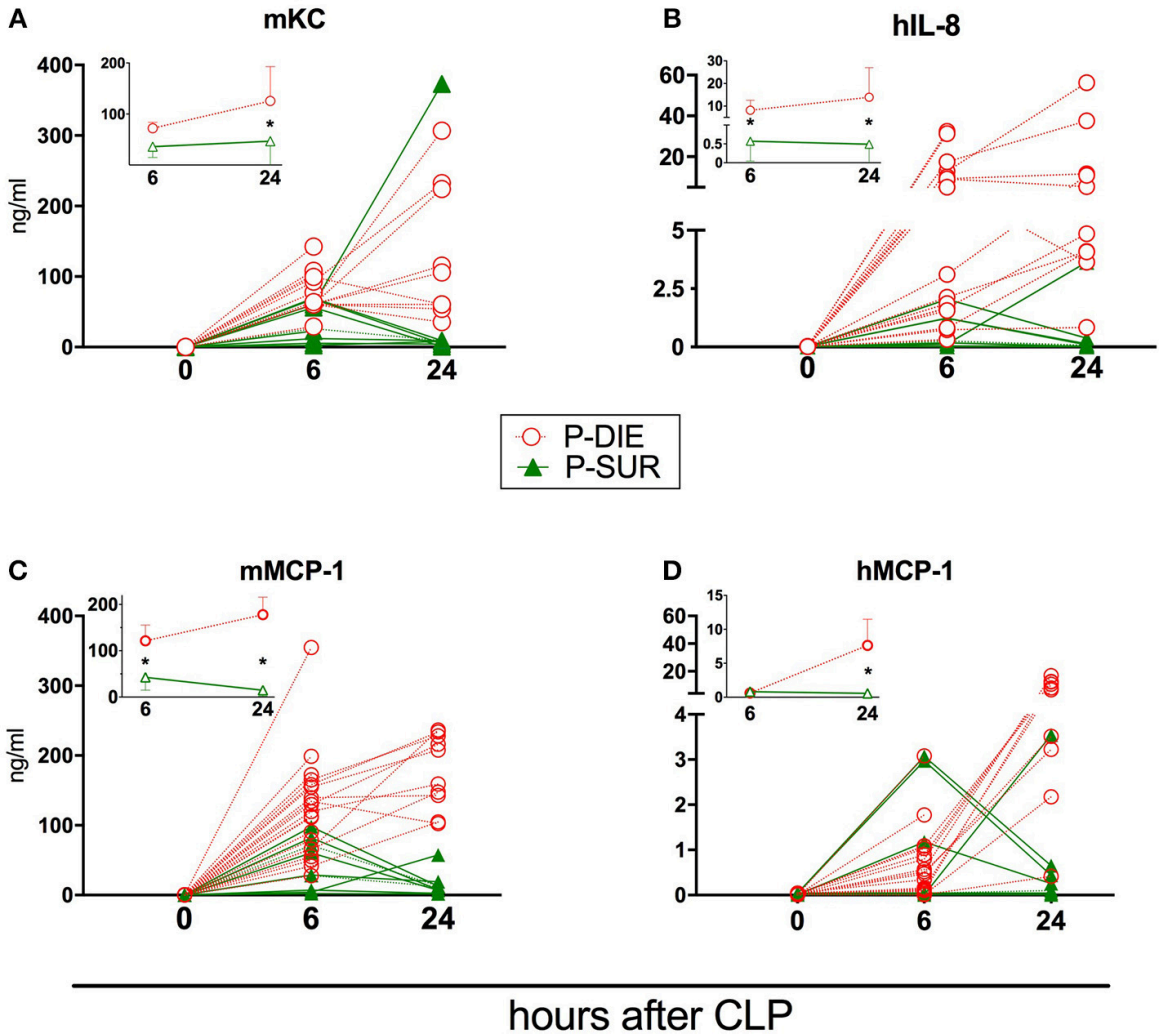
Comparison of human and murine chemokines depending on the post-CLP outcome in humanized mice. Insets present data as mean values and 95% confidence interval bars. **(A)** murine KC; **(B)** human IL-8; **(C)** murine MCP-1; **(D)** human MCP-1. (P-DIE *n* = 22, P-SUR *n* = 9). Concentrations of chemokines between P-SUR and P-DIE mice were compared by the *t*-test at each time-point separately. *p < 0.05.

**Figure 6:**
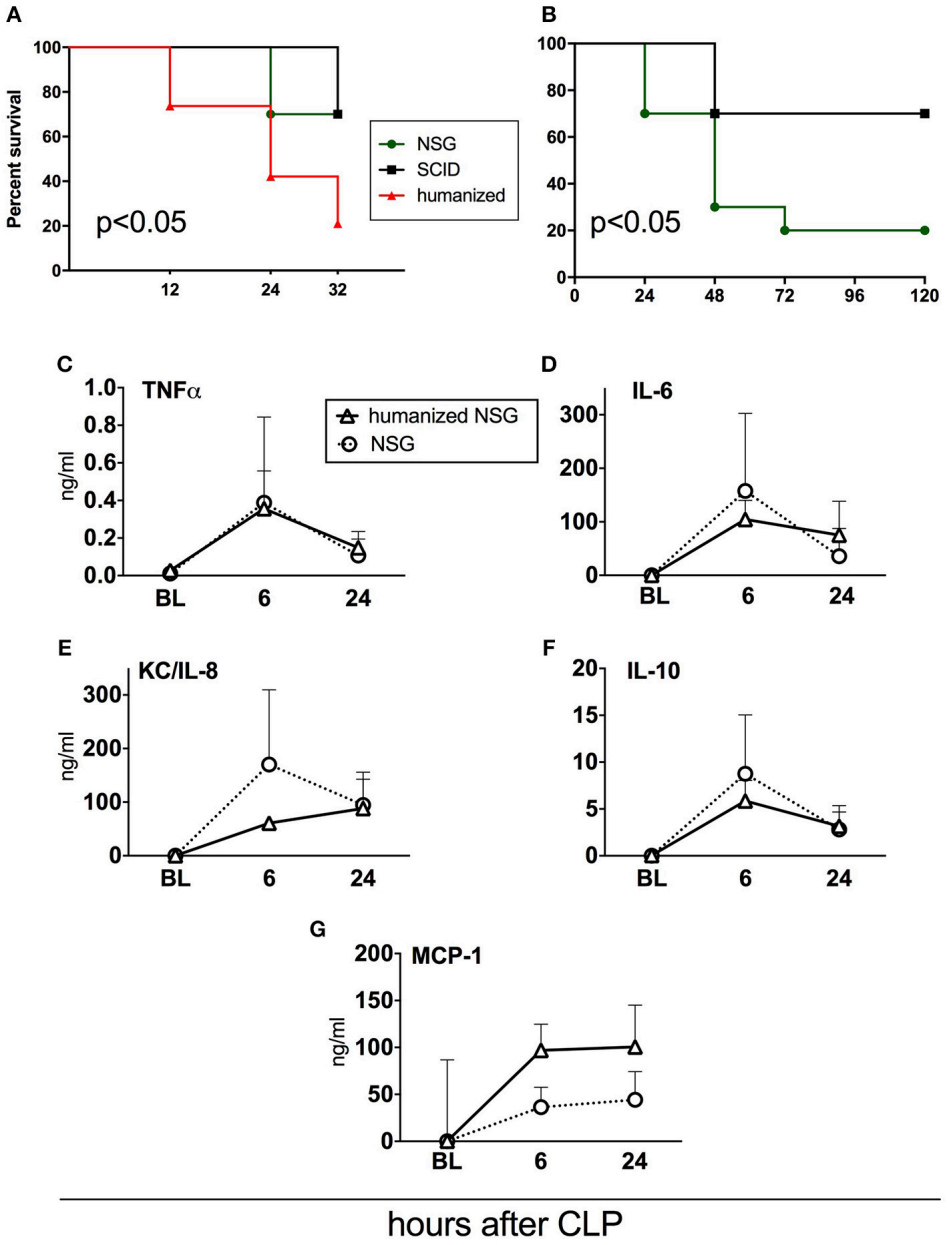
Survival and cytokine responses in humanized NSG vs. naïve NSG and SCID mice. All mice were subjected to CLP with 25G needle size. **(A)** Acute survival curves of hNSG (*n* = 19), naïve NSG (*n* = 10), and SCID (*n* = 10). Humanized NSG mice were terminated at 32 h post-CLP for tissue collection and analysis. Survival curves were compared by Log-rank test, *p* < 0.05 for hNSG vs. NSG and vs SCID. **(B)** Long-term survival curve of NSG and SCID mice from the same experiment. (NSG *n* = 10, SCID *n* = 10, hNSG *n* = 19). Survival curves were compared by Log-rank test, *p* < 0.05. Murine cytokine responses in NSG (*n* = 10) and hNSG (*n* = 19) G: **(C)** TNF; **(D)** IL-6; **(E)** KC/IL-8; **(F)** IL-10; **(G)** MCP-1. Murine cytokine levels in humanized vs. NSG mice were compared by the paired *t*-test at each time-point separately.

Two CLP runs were performed in humanized mice: (a) with a 25G needle (*n* = 19) and (b) with a 27G needle (*n* = 14). A single CLP run with a 25G needle was performed in NSG (*n* = 10) and SCID (*n* = 10) mice. Humanized mice from both runs were combined for data shown in Figures 1–5 (i.e., outcome-based comparisons). Two mice that died within 6 h of CLP were excluded as death was deemed to be due to the fact that they did not recover from the anesthesia/surgical intervention itself (and not sepsis). For the comparison of NSG to SCID in Figure 6 (i.e., comparison of sensitivity to an identical insult), humanized mice from the first run (25G needle) only were used. Based on our previous CLP protocols (21, 28), we used two needle sizes in order to introduce more longitudinal outcome variability into the study. In the current study, both sizes produced similar overall outcomes at 32 h (Supplementary Figure 1). The more divergent variability is advantageous given that our study focused on the outcome-based differences (i.e., surviving vs. moribund phenotype) and not a CLP phenotype produced by a specific needle size. A similar (2-needle) approach was recently used by Kim et al. (29) in testing a hydrocortisone/ascorbic acid/thiamine treatment in mouse CLP.

From 2 h post-CLP on, all mice received subcutaneous broad spectrum antibiotic therapy (25 mg/kg imipenem, Zienam® MSD, Lucerne, Switzerland) and fluid resuscitation (1 ml Ringer's solution) with analgesia (0.05 mg/kg buprenorphine, Bupaq® Richter Pharma, Austria) twice daily (approximately every 12 h) for 5 consecutive days post-CLP.

#### Monitoring and Prediction of Outcome

All CLP mice were not maintained in a large animal facility but were kept in a small animal room to enable close observation for implementation of the humane endpoints and to allow allocation of individual mice into either predicted-to-die (P-DIE) or predicted-to-survive (P-SUR) group. All mice were monitored for clinical signs of illness and their status was evaluated using a modified mouse clinical assessment scoring system [M-CASS; e.g., fur, posture, mobility, alertness, startle, and righting reflex (22)] starting 12 h post-CLP. Also, rectal temperature was monitored (Fluke Series II thermometer, Fluke USA) at least twice daily (or more often whenever a mouse deteriorated). Mice were deemed moribund and assigned as P-DIE whenever the righting reflex was absent or/and M-CASS score ≥8 and immediately euthanized. Following the previously used protocol (21, 30), two P-SUR mice were randomly selected and euthanized at 24 h to serve as a time-matched comparison for moribund mice sacrificed as P-DIE at 24 h post-CLP. Body temperature recordings served as a supportive measure in the general health assessment and euthanasia decision-making scheme (and the P-DIE vs. P-SUR allocation) given that the body temperature fluctuations were not previously validated to serve as a predictor of outcome in any humanized mice disease model. Two mice (out of 33) died of post-surgical complications within 5 h of CLP and were excluded from data analysis.

### Flow Cytometry

Flow cytometry was used for phenotyping of engrafted human cells. Probes of blood or re-suspended cells retrieved from solid organs were incubated with inactivated mouse serum to block unspecific Fc receptors. Then, mixtures of monoclonal antibodies against human antigens were added and cells were incubated for 30 min at room temperature. The following antibodies were used: anti-CD45 AmCyan, anti-CD14 PE, anti-CD3 Pacific Blue, anti-CD4 APC, anti-CD8 PE, anti-CD33 PeCy5.5, anti-HLA-DR PE.CY7, (BD Bioscience), anti-CD80 Alexa Fluor 488 (Biolegend, San Diego, USA). After staining, erythrocytes were lysed with BD Pharm Lyse solution (BD Biosciences) for 10 min, washed with PBS with 2% Newborn Calf Serum (NCS), and re-suspended in 0.5% paraformaldehyde in PBS. Cells were acquired using the FACS Canto II flow cytometer and Diva software (BD Bioscience, San Jose, CA). Analysis of immunophenotype was carried out applying the FlowJo 10 software (TreeStar, Inc., now part of FlowJo LLC, Ashland, OR, USA). The applied anti-human antibodies were verified for cross-reactivity with cells of non-transplanted mice and no staining was present.

### Cytokine Measurements

Facial vein blood samples were obtained at baseline, 6 and 24 h after CLP as described (31). Samples were stored at −86°C until analysis. Plasma concentration of human and murine cytokines (IL-6, IL-8/KC, IL-10, TNF, MCP-1) was measured using Luminex Multiplex Immunoassay (Invitrogen, Thermo Fisher Scientific, Vienna, Austria) according to the manufacturer's protocol. The cross-reactivity rates provided by the supplier (expressed as % recovery of either human or mouse protein) for mouse targets are as follows: IL-6: 0.1%; TNFα: 0.0%; MCP-1: 0.5%; IL-10: 0.2% Gro alpha KC: 0.1%. For human targets: IL-10: 0.0%; IL-6: 0.0%; IL-8: n.a.; Gro alpha: 0.0%; MCP-1: not checked; TNFα: 0.0%. Two P-SUR mice were also sacrificed at 24 h to serve as comparisons to P-DIE that were sacrificed at the same time-point. Additionally, in Figures 2, 6, the individual cytokine values measured at 24 and 32 h post-CLP were merged (for P-DIE and P-SUR, respectively) and presented as the 24 h time-point only. This was justified by an overlapping cytokine expression (in all cytokines) between those two time-points (i.e., *p* > 0.1 for 24 vs. 32 h difference; data not shown).

### Statistical Analysis

Normality of all data sets was assessed using the Shapiro-Wilk test and log-transformed to eliminate skewness and/or non-parametric distribution whenever present. Data are expressed as means and 95% confidence intervals (CI) if not otherwise stated. Comparisons between P-DIE and P-SUR group were performed by *t*-test (with Welch correction for unequal variances if needed) given that the P-DIE group did not meet assumptions (non-random deaths) for repeated measures testing. The correlation strength between the variables was assessed using the Pearson's rank correlation coefficient. Receiver operator curves (ROC) were calculated for the evaluation of predictive utility of selected variables. Cut-off values were chosen using the Youden's index. The areas under the curves (AUC) with CIs were calculated for assessment of the accuracy of the test. Sensitivity and specificity were calculated for the selected cut-off values of the variables. The Kaplan-Meier survival curves were compared using the log-rank test. *p* < 0.05 was considered significant. Statistica 12 (StatSoft, Inc., USA) and GraphPad Prism 5 (GraphPad, Inc., USA) softwares were used for evaluating the statistical significance and/or graphical depiction of the data.

## Results

### Development of Humanized Mice

Humanized NSG mice were generated based on our previously used protocol (17). The busulfan myeloablation regimen was proven to be efficient and safe without any clinical pathology in the recipient mice. Development of human granulocytes, monocytes, and B and T cells was confirmed to be similar to our previously reported readouts in the spleen, bone marrow, and peripheral blood (17) (Supplementary Table 1).

Among circulating human CD45+ cells, CD20+ B cells, and CD33+ myeloid cells were the most frequent. Analysis of the thymus revealed the development of human CD3+ cells in the mouse host (data not shown). Chimerism of human CD45+ (hCD45+) cells in the peripheral blood was confirmed in all transplanted mice 1 week before the CLP experiments [mean 19.6% 95CI (11.3–27.1)]. Furthermore, all humanized mice enrolled in the study had >18% of hCD45+ cells in the bone marrow (retrospective post-CLP verification; Supplementary Table 2).

### Humanized Septic Mice: Prediction of Outcome

CLP surgery in humanized mice resulted in 55% mortality at 24 h and 71% at 32 h (the experiment termination time-point) (Figure 1). At the 32 h time-point, all remaining mice were assigned either to the predicted-to-die (P-DIE; *n* = 22) or predicted-to-survive (P-SUR; *n* = 9) group based on the clinical assessment and body temperature readouts (see Methods) for further retrospective comparisons of the measured parameters.

We also performed a retrospective ROC analysis using body temperature recordings only (taken at 6 h post-CLP) to assess whether a prediction of (short term) outcome is possible in humanized septic mice. This analysis combined all CLP mice and the outcome was based on our clinical P-DIE/P-SUR allocation. The area under the curve reached 0.99 (95CI: 0.98–1.00) with the cut-off set at 33.2°C (i.e., 100% sensitivity, 95% specificity for the next 32 h). Of note, all P-SUR mice (i.e., allocated at 32 h post-CLP) had body temperature of at least 33.2°C or higher (Supplementary Figure 2).

### Humanized Septic Mice: Outcome-Based Characterization of Human Monocytes From the Bone Marrow and Spleen

To investigate the cellular response of humanized mice to abdominal sepsis, we analyzed CLP-induced leukocyte changes in two compartments: (a) the bone marrow and (b) spleen. Leukocytes from P-DIE mice were harvested between 24 and 32 h after CLP (*n* = 11) in order to also harvest cells from mice that became moribund prior to the end of the study. Leukocytes from remaining P-SUR mice were collected at the termination of the experiment (i.e., 32 h post-CLP; *n* = 9).

There was a similar percentage frequency of hCD45+ leukocytes in both compartments: 34% P-DIE vs. 45% P-SUR in the bone marrow and 18 vs. 26% in the spleen (Table 1). In the bone marrow, hCD14+ monocytes (Figures 2A,D) were similar regardless of outcome. In contrast, the splenic hCD14+ monocytes were 3-fold higher in P-DIE compared to P-SUR mice (Figure 2G).

**Table 1:** Human immune cells in the humanized mice based on the CLP outcome.

**Cell type**	**P-SUR (*n* = 9)**	**P-DIE (*n* = 11)**	***p***
**BONE MARROW**			
hCD45+ [%]	45.14 (27.39–62.88)	34.82 (20.26–49.38)	>0.05
hCD14+ [%]	21.16 (−4.16–46.47)	27.30 (9.29–45.31)	>0.05
hCD14+HLA-DR [GMF]	406 (−12.00–824.30)	321.60 (213.40–429.90)	>0.05
hCD14+CD80 [GMF]	797.00 (566.90–1027.00)	546.40 (394.70–698.20)	**<0.05**
**SPLEEN**			
hCD45+ [%]	26.23 (6.05–46.41)	17.57 (7.95–27.19)	>0.05
hCD14+ [%]	2.91 (−0.06–5.87)	8.48 (4.27–12.69)	**<0.05**
hCD14+HLA-DR [GMF]	406.20 (−12.00–824.30)	321.60 (213.40–429.90)	>0.05
hCD14+CD80 [GMF]	835.40 (612.90–1058.00)	492.60 (337.60–647.70)	**<0.01**

*Statistically significant values are bolded*.

We also evaluated the geometric median fluorescence (GMF) of anti-HLA-DR and anti-hCD80 antibodies on human monocytes (Figures 2B,C). The HLA-DR GMF on the bone marrow monocytes was similar, irrespective of outcome (Figure 2E). In contrast, the GMF of CD80 was decreased by 32% on the human monocytes from P-DIE (vs. P-SUR) mice (Figure 2F).

The intensity of anti-HLA-DR staining of the splenic monocytes did not differ between the groups (Figure 2H), however, the GMF of anti-hCD80 was 1.3-fold higher in the P-SUR compared to P-DIE mice (Figure 2I). Of note, the frequency of the hCD3+ T cells appeared to be (4-fold) higher in the spleen of P-SUR than P-DIE mice but this difference was not statistically significant (high individual variability).

Pearson‘s rank correlation coefficient revealed a moderate, but significant correlation between the frequency of hCD3+ T cells in the spleen and the hCD80 GMF on hCD14+ monocytes (*r* = 0.62, *p* < 0.01).

### Humanized Septic Mice: Comparison of the Human vs. Murine Circulating Cytokines

Repetitive small-volume blood sampling enabled us to sequentially evaluate the concentration of cytokines in septic mice without sacrificing them. First, we compared the combined kinetics of all measured circulating cytokines and chemokines of murine and human origin. Irrespective of outcome, the analyzed mediators typically peaked at 6 h and decreased at 24 h post-CLP (Figure 3).

Next, we assessed the total content of circulating cytokines of human and murine origin. The combined human mediators reached 24% of all measured cytokines in healthy mice, 8% at 6 h and 7% at 24 h post-CLP in septic mice (Figure 3A). Accordingly, the post-CLP concentration of individual human mediators was, in most cases, an order of magnitude lower than of their murine counterparts. Interestingly, human TNFα was the only cytokine whose early post-CLP peak (i.e., 6 h) was higher (by 7-fold) than the murine TNFα (Figure 3B).

While the post-CLP response dynamics of human and murine TNFα, IL-6, and IL-8/KC were similar (Figures 3B,C,E), circulating human IL-10 and MCP-1 showed a delayed increase in comparison to the murine mediators (Figures 3D,F).

There was a significant negative correlation between expression of CD80 on splenic human monocytes and the concentration of human systemic pro-inflammatory cytokines, while no correlation with IL-10 was observed 6 h after CLP (Supplementary Figure 3).

### Humanized Septic Mice: Comparison of Humanized and Murine Circulating Cytokines Based on Outcome

Regardless of the origin species, the concentration of all circulating cytokines (except murine KC, human IL-10, and MCP-1) was typically higher in P-DIE compared to P-SUR mice at 6 h post-CLP (Figures 4, 5). At 24 h, this outcome difference remained (or appeared) in IL-6, IL-8, IL-10, and MCP-1 (both origins) and disappeared in TNFα (both origins) and KC (i.e., mouse only).

Furthermore, the different species-dependent IL-10 and MCP-1 dynamic (observed in Figures 3D,F) was predominantly due to the changes in the P-DIE mice (IL-10: Figures 4E,F; MCP-1: Figures 5C,D); the release of human mediators was delayed to 24 h post-CLP compared to the immediate rise of the mouse cytokines at 6 h.

We also performed the ROC analysis to assess the prognostic outcome accuracy of the mediators measured at 6 h post-CLP. For murine cytokines, TNFα and IL-6 had the highest AUC of 0.93 and 0.97 (Table 2). Of note, AUC for human IL-8 reached 0.93, indicating a strong discriminative value for this human chemokine.

**Table 2:** Prognostic accuracy of human and murine cytokines 6 h post-CLP for 24 h death prediction.

**Cytokine**	**AUC (95CI) for murine**	**AUC (95 CI) for human**
TNF	0.93 (0.85–1.00)	0.84 (0.68–1.00)
IL-6	0.97 (0.90–1.00)	0.85 (0.70–1.00)
IL-10	0.92 (0.81–1.00)	0.72 (0.54–0.90)
IL-8/KC	0.80 (0.60–1.00)	0.93 (0.83–1.00)
MCP-1	0.85 (0.70–0.99)	0.61 (0.34–0.88)

*AUC, area under the curve; CI, confidence interval*.

### Humanized vs. Immunodeficient Mice: Outcome

To verify how human immune cells transplanted to immuno-compromised NSG mice influence sepsis phenotype, we simultaneously performed CLP in naïve NSG and SCID mice (Figure 6). We also included SCID mice as this strain is less immunodeficient than NSG; its adaptive (but not innate) immunity is impaired (32). Using the same CLP protocol, we observed that the mortality of both non-humanized strains was lower by 46% compared to the humanized NSG mice (33 vs. 79%; *p* < 0.05) at 32 h after CLP (Figure 6A). Given that we did not collect tissues from the non-humanized mice, NSG and SCID animals were followed up until day 5. Compared to SCID, NSG mice were more vulnerable to the identical CLP insult (30 vs. 80% mortality; *p* < 0.05; Figure 6B).

### Humanized vs. Immunodeficient Mice: Circulating Cytokines

To further evaluate the impact of the human cells on murine humoral inflammatory response itself, we compared the concentrations of circulating murine cytokines after an identical CLP insult in the naïve and humanized NSG mice. The pattern of the post-CLP cytokine release was generally similar regardless of the xenotransplantation of the human immune cells (Figures 6C–G). The trend for an enhanced MCP-1 release (Figure 6G) after humanization did not reach statistical significance.

## Discussion

The development of humanized mice provides a unique opportunity to study responses of human immunocompetent cells residing in different tissues in a controlled setting in sepsis, however, such a model requires an extensive characterization of human and murine immuno-inflammatory components. In our study, we used clinically-relevant CLP sepsis (26) followed by antibiotic-, fluid-, and analgesic treatment to recapitulate septic patient care. The findings show for the first time that both human and murine cytokine responses have similar dynamics with regard to acute (early) post-CLP outcome. Moreover, we demonstrated that an early release of human TNFα exceeded by 10-fold the release of its murine counterpart. We also observed that the cytokine response correlated with human monocyte changes, suggesting dynamic interactions in the human immune compartment. Importantly, we revealed that the applied protocol of human HSC transplantation did not impair the ability of murine cells to mount an inflammatory response to sepsis.

We chose to focus on the response of human monocytes in the spleen and bone marrow as these are sites of robust immune processes during infection. The high frequency of monocytes among human cells in the bone marrow likely resulted from selective mobilization of other cell types to the circulation. The number of human monocytes in the spleen of P-SUR was diminished compared to P-DIE mice. CLP sepsis evokes apoptosis of human cells in the humanized mice similar to the phenomenon observed in septic patients (15, 16). Different responses of monocytes at these two sites can be attributed to the local tissue microenvironment and local production of monocytes in the bone marrow (33). We observed a significant drop of HLA-DR and CD80 expression on monocytes from humanized mice post-CLP but no difference in HLA-DR expression was apparent between P-DIE and P-SUR mice. The lack of difference in HLA-DR expression between groups of opposing outcome was surprising; HLA-DR decrease was shown to be a marker of immunosuppression and it correlated with the magnitude of inflammatory response (34). In line with our findings, however, it has been demonstrated that changes in the HLA-DR expression did not constitute an accurate marker of mortality in septic shock patients (35, 36).

CD80 is one of the co-stimulatory molecules required for T-cell activation by antigen-presenting cells. Nolan et al (29) demonstrated an increased CD80 expression on the circulating monocytes in septic patients and peritoneal macrophages from septic mice (37). Moreover, CD80^−/−^ mice had improved survival after CLP (38) and in another study, CLP mice lacking CD80 displayed lower circulating IL-6 levels (38). The above was contrasted by our data: (a) Monocytes from P-DIE humanized mice showed a greater reduction of CD80 expression in comparison to their P-SUR littermates and (b) we observed a negative correlation between the CD80 expression and circulating cytokine concentration. There are several possible reasons for these discrepancies. First, we analyzed CD80 expression on the bone marrow and splenic monocytes, while the other studies focused on the circulating and peritoneal cells. The choice of sampling sites seems to be of vital importance as the immune response in sepsis appears to be highly compartmentalized (39, 40). Second, monocytes from humanized mice were shown to be partly immature, therefore their response to infection can differ from the mature cells (41). Notably, Gille et al. did not find expression of CD80 on monocytes from humanized mice developed on newborn NSG mice transplanted with UCB cells (41). Third, our humanized mice showed a low frequency of human T cells which interacted with monocytes, therefore, the mutual activation of these cells may differ from the normal human setting. Finally, the interspecies interactions in humanized mice are not known and it cannot be ruled out that the murine microenvironment modulates selected responses of human cells. It would be of interest to examine the expression of the interleukin-1 receptor-associated kinase-M in the monocytes of post-CLP humanized mice as this molecule is a master-regulator of CD80 surface expression (38).

We applied small-volume repetitive sampling (31) to monitor the changes of human and murine cytokines in early sepsis. Previously we have demonstrated a simultaneous release of pro-and anti-inflammatory cytokines in the early post-CLP phase (19). Both at baseline and post-CLP, the concentration of measured murine cytokines outbalanced human cytokines. Both human and murine cytokines were upregulated 6 h post-CLP, yet, it was apparent that murine circulating cytokines predominate (concentration-wise) over the human mediators in an approximate 9:1 ratio. This has never been reported before but such a magnitude of discrepancy is not surprising given that the only source of human circulating cytokines in these mice were hematopoietic cells. In contrast, despite humanization, mice maintain several other murine-origin cell types known to secrete cytokines (e.g., endothelium, hepatocytes, myocytes). Most murine cytokines are not active on human cells while some human cytokines are not species-specific (42). The negative correlation between expression of CD80 on human monocytes and concentration of human cytokines suggests that humanized mice recapitulate systemic interactions between humoral and cellular elements of human immunity. Interestingly, there was a robust early peak of human TNFα which markedly outbalanced murine TNFα. As human TNFα cross-reacts with murine receptors (43), it can be speculated that the combined high TNFα load contributes to the increased mortality of humanized mice. The RIP3-mediated induction of necroptosis was shown by Duprez et al. (44) to contribute significantly to early deaths after CLP. We hypothesize that this mechanism was responsible for a greater sensitivity to CLP of humanized mice compared to naïve NSG or SCID mice to CLP. Similarly to our results, humanized mice were shown to be more sensitive to *S. aureus* infection and CLP than NSG or wild-type mice (16, 45). Ye et al. (16) showed that the high mortality of humanized mice after CLP was related to the production of HMGB-1 by human myeloid cells. It cannot be ruled out that the process of xenotransplantation impairs the murine granulocytic response in humanized mice that is otherwise efficient in the naïve NSG mice. Additionally, it is apparent that humanization did not change the general profile of post-CLP cytokine response in NSG mice. Therefore, the tissues/organs were subjected to the murine inflammatory response, preserving the ability of the host to develop organ dysfunction (recapitulating clinical sequelae).

Next, we examined whether the disease severity modulated the human/murine cytokine response. The relatively high severity (i.e., 71% by 32 h post-CLP) that we employed in our study was dictated by two elements: (a) we sought to provoke robust enough post-CLP changes in both compartments (i.e., human and murine) to enable their effective comparison and (b) a naturally high sensitivity of humanized mice to infection/injury. As early as 6 h post-CLP, all human and murine cytokines (except for human IL-10 and MCP-1) were markedly elevated in the P-DIE mice compared to P-SUR. At 24 h, only the concentration of human/murine TNFα dropped to a similarly low level regardless of outcome. Clinical sepsis data regarding the relationship between TNFα concentration and sepsis mortality are conflicting. However, several studies, in line with our own observations, demonstrated that early TNFα release (i.e., measured close to the onset of sepsis) correlates with mortality (46–48). Importantly, in septic patients, the kinetics of the TNFα response largely vary depending on patient characteristics (47); our CLP model (by default) is much more controlled, and features a relatively steady response. As revealed by the ROC analysis, human cytokines that showed the best predictive value for mortality were IL-8 and IL-6, which is in line with the clinical data (49). However, murine IL-6 and TNFα were characterized by even higher AUC values. This is rational as these cytokines are also produced by non-hematopoietic cells which constitute the majority of tissues in humanized mice.

This study has several limitations. First, our humanized mice show a (human) chimeric heterogeneity. This is unavoidable as chimerism largely depends on the donor (50). However, the heterogeneity of donors partly recapitulates the genetic variability that is present in the clinical scenario. Second, we did not further investigate human monocytes to explain the post-CLP discrepancy in CD80 expression. Third, our model included only a single gender and was characterized by a relatively high CLP severity; the current study should be repeated in males and in a low-severity sepsis scenario. Also, a study in aged humanized mice should be performed as a next step to mimic demographic characteristics of septic patients. Additionally, our study failed to characterize the fluctuations beyond day 2 post-CLP; an assessment of cellular and humoral inflammatory changes in the chronic CLP phase should be attempted. Finally, the body temperature-based prediction of early CLP outcome needs to be verified prospectively given that our assessment used retrospective readouts.

Summarizing, this study characterizes for the first time an outcome-dependent evolution of cellular (i.e., bone marrow and splenic monocytes) and circulating cytokine responses in humanized mice. We demonstrate that humanized mice subjected to CLP recapitulate important inflammatory features of clinical sepsis supporting the notion that this model can be utilized in preclinical sepsis research to maximize its translation potential.

## Data Availability

The raw data supporting the conclusions of this manuscript will be made available by the authors, without undue reservation, to any qualified researcher.

## Ethics Statement

This study was carried out in accordance with the recommendations of ARRIVE guidelines. The protocol was approved by the Local Ethic Committee no IV in Warsaw, Poland.

## Author Contributions

TS: design, performing experiment, analysis of the study, and writing manuscript. SD: study design, performing experiment, and correction of manuscript. GH: study design and performing experiments. MJ: sample analysis. KS: cord blood preparation. ZP: cord blood preparation and correction of manuscript. JK: study design and correction of manuscript. MO: study design, performing experiments, data analysis/interpretation, and writing manuscript.

### Conflict of Interest Statement

The authors declare that the research was conducted in the absence of any commercial or financial relationships that could be construed as a potential conflict of interest.
