# Supplementary material for: The Fluctuations of Leukocytes and Circulating Cytokines in Septic Humanized Mice Vary With Outcome
Source: Front Immunol. 2019 Jun 26;10:1427. doi: 10.3389/fimmu.2019.01427 (PMC6607920; doi:10.3389/fimmu.2019.01427)
Supplement: Supplementary file 1 [file Data_Sheet_1.docx]

**ONLINE SUPPLEMENT**

**The Fluctuations of Leukocytes and Circulating Cytokines in Septic Humanized Mice Vary with Outcome.**

Tomasz Skirecki^1^, Susanne Drechsler^2^, Grażyna Hoser^1^, Mohammad Jafarmadar^2^, Katarzyna Siennicka^3^, Zygmunt Pojda^3^, Jerzy Kawiak^1^, Marcin F. Osuchowski^2*^

^1^ Laboratory of Flow Cytometry, Centre of Postgraduate Medical Education, Warsaw, Poland

^2^Ludwig Boltzmann Institute for Experimental and Clinical Traumatology in the AUVA Research Center, Vienna, Austria

^3^Department of Cell Engineering, Maria Sklodowska-Curie Memorial Cancer Center and Institute of Oncology, Warsaw, Poland

**Supplemental Table 1**. **Human immune cells development eight weeks after transplantation of the umbilical cord blood CD34+ cells.**

| Lineage | Bone marrow [median] | Spleen  [median] | Peripheral blood  [median] |
| --- | --- | --- | --- |
| CD20+ B cells [%] | 35.5 | 24.6 | 38.5 |
| CD3+ T cells [%] | 1.1 | 6.7 | 1.4 |
| CD14+ monocytes [%] | 28.7 | 14.2 | 4.9 |

**Supplemental Table 2. Frequency of human CD45+ cells in the bone marrow of humanized mice sacrificed after CLP.**

| **Mouse** | **BM hCD45 [%]** |
| --- | --- |
| **1** | 56.6 |
| **3** | 23.9 |
| **4** | 18.3 |
| **6** | 42.6 |
| **7** | 33.6 |
| **9** | 53.9 |
| **11** | 55.1 |
| **12** | 39.8 |
| **13** | 40.7 |
| **14** | 53.7 |
| **15** | 18.9 |
| **19** | 68.6 |
| **23** | 27.3 |
| **24** | 25.4 |
| **26** | 35.4 |
| **27** | 64.4 |
| **28** | 82.8 |
| **29** | 19.9 |
| **30** | 26.6 |

**Supplemental Figure 1. Survival graphs of two CLP runs on humanized NSG mice.**

**Supplemental Figure 2. Comparison of all body temperature measurements in P-DIE and P-SUR mice.**

**Supplemental Figure 3. Correlation coeffcients of human cytokines 6 hours after CLP with the expression of CD80 on human splenic monocytes at the time of death.** Concentration of human plasma cytokines was correlated with the geometric mean fluorescence (GMF) of anti-CD80 staining of human monocytes in the spleens of septic mice (24-32 hours after CLP). Pearson correlation values are marked on the graphs. A. CD80 GMF vs TNF; B. CD80 GMF vs IL-6; C. CD80 GMF vs IL-8; D. CD80 GMF vs IL-10; E. CD80 GMF vs MCP-1.
